# Middle Meningeal Artery Embolization versus Surgery in Patients with Chronic Subdural Hematoma—No More Fence Sitting?

**DOI:** 10.3390/neurolint15040096

**Published:** 2023-12-06

**Authors:** Dalibor Sila, Francisco Luis Casnati, Mária Vojtková, Philipp Kirsch, Stefan Rath, František Charvát

**Affiliations:** 1 Department of Neurosurgery and Interventional Neuroradiology, Donau Isar Klinikum Deggendorf, Perlasberger Str. 41, 94469 Deggendorf, Germany; francisco-luis.casnati@donau-isar-klinikum.de (F.L.C.); stefan.rath@donau-isar-klinikum.de (S.R.); 2 Department of Neurosurgery and Neurooncology, First Faculty of Medicine, Charles University in Prague, Military University Hospital, U Vojenské Nemocnice 1200, 16902 Praha, Czech Republic; frantisek.charvat@uvn.cz; 3 Department of Statistics, Faculty of Economic Informatics, University of Economics in Bratislava, Dolnozemská Cesta 1/b, Bratislava 85235, Slovakia; maria.vojtkova@euba.sk; 4 Department of Radiology and Interventional Radiology, Donau Isar Klinikum Deggendorf, Perlasberger Str. 41, 94469 Deggendorf, Germany; philipp.kirsch@donau-isar-klinikum.de

**Keywords:** middle meningeal artery, subdural hematoma, embolization, endovascular treatment, surgery, burr hole trepanation, craniotomy, treatment failure

## Abstract

Background: Endovascular treatment of patients with chronic subdural hematoma using middle meningeal artery (MMA) embolization could become an alternative to surgical hematoma evacuation. The aim of the study was to compare methods and identify parameters to help determine the correct treatment modality. Methods: We retrospectively reviewed 142 cases conducted internally; 78 were treated surgically and 64 were treated using MMA embolization. We analyzed the treatment failure rate and complications, and using a binary logistic regression model, we identified treatment failure risk factors. Results: We found a comparable treatment failure rate of 23.1% for the surgery group and 21.9% for the MMA embolization group. However, in the MMA embolization group, 11 cases showed treatment failure due to early neurological worsening with a need for concomitant surgery. We also found a recurrence of hematoma in 15.4% of cases in the surgery group and 6.3% of cases in the MMA embolization group. Conclusion: Both modalities have their advantages; however, correct identification is crucial for treatment success. According to our findings, hematomas with a maximal width of <18 mm, a midline shift of <5 mm, and no acute or subacute (hyperdense) hematoma could be treated with MMA embolization. Hematomas with a maximal width of >18 mm, a midline shift of >5 mm, and no membranous segmentation could have better outcomes after surgical treatment.

## 1. Introduction

Chronic subdural hematomas (CSDHs) occur in about 20 per 100,000 persons per year and are more commonly encountered in the elderly population [1]. The pathophysiological cascade leading to the formation and expansion of CSDHs includes traumatic mechanisms followed by inflammatory processes leading to the formation of vascularized permeable membranes. Cortical atrophy appears to be a triggering factor activating a cascade of transendothelial cellular inflammation, membrane formation, and neovascularization leading to CSDH formation [2]. A widely performed surgical treatment uses burr hole trepanation or craniotomy that targets hematoma evacuation. This treatment has very little or no influence on the secretion phase of the disease and further hematoma expansion, which could be the main reason for the recurrence rate reaching up to 33% after surgical treatment [3,4,5]. The CSDH recurrence rate after surgery can be influenced by the use of antithrombotic therapy (AT). On the other hand, discontinuation of AT can increase the risk of thrombotic events, especially in the early phase after surgery [6]. The next treatment option is conservative therapy, which could be successful in patients with either mild or no symptoms, low hematoma density, and the absence of paresis [7]. In these cases, observation could be an option [8]. The usage of steroids in the treatment of CSDHs remains a controversial topic [9]. Steroids can reduce the recurrence rate, but may have no influence on treatment success or mortality rate, and may significantly increase the risk of adverse events [10].

Since Mandai (2000) first described a case of middle meningeal artery (MMA) embolization [11], many studies followed [4,12,13,14,15]. The main challenge is the correct treatment modality selection; however, the indication criteria for endovascular treatment of CSDHs are not clear. We decided to analyze our data from patients treated at the Neurosurgical and Neurointerventional Department at Donau Isar Hospital in Deggendorf (Germany) in 2020 and 2021 and compare surgical and endovascular treatments. The primary outcome we identified was the treatment failure rate, which we defined as a need for a second hematoma treatment due to neurological deterioration. Secondary outcomes were treatment-related complications, recurrence rate, the number of follow-up CT scans, the duration of follow-up measured in days, and the types of treatment complications patients experienced. The next goal was to identify factors that increased the treatment failure risk of both treatment modalities. 

## 2. Materials and Methods

### 2.1. Retrospective Registration and Inclusion Criteria

The review included adult patients with a unilateral or bilateral chronic subdural hematoma seen in an admission CT scan. Patient age, gender, anticoagulation and/or antiplatelet medication in patient history, potential prior hematoma treatment, and site of hematoma were recorded. We analyzed the hematoma maximal width measured in the frontal plane taken by CT scan, midline shift (the membranous segmentation of the hematoma), and hyperdense acute or subacute parts of the hematoma. Neurological status at patient admission was documented using a National Institutes of Health Stroke Scale (NIHSS) [16] score, and the patient symptoms were grouped by major symptoms—hemiparesis, dysphasia, decreased consciousness; minor symptoms (e.g., headache, vertigo, dizziness); and no symptoms. The analysis of admission and control CT scans was performed by an unbiased radiologist (PK).

### 2.2. Surgical and Endovascular Procedures 

All treatments were performed under general anesthesia. A burr hole trepanation or small craniotomy approach was used for the surgical evacuation of a CSDH. After the incision of the dura mater and hematoma evacuation, we irrigated the subdural space with Ringer solution and placed a closed drainage system in the subdural space. After a follow-up CT scan, the drainage system was removed on day three.

The goal of the endovascular procedure was to perform the MMA embolization. After puncturing the common femoral artery, a 4F sheath was inserted. The 4F vertebral catheter was delivered to the external carotid artery proximal to the maxillary artery exit. Using a roadmap technique, a Progreat^®^ microcatheter and microwire (Terumo Medical Corporation, Somerset, NJ, USA) were placed into the MMA. For embolization of the MMA, we used Contour^TM^ 150–250 µm polyvinyl alcohol (PVA) particles (Boston Scientific, Marlborough, MA, USA) with contrast solution. The artery embolization was performed using a roadmap technique. After the treatment, the patients were observed in the intermediate care unit for 72 h. The follow-up CT scan was performed on day three. 

### 2.3. Statistical Analysis

Statistical analysis was performed using SPSS version 22.0 (SPSS Inc., Chicago, IL, USA). The independent sample T-test was used to determine differences in numeric mean scores, the Mann–Whitney U test was performed to determine differences in the median scores, the χ^2^-test was used to determine differences in the frequency distribution, and Fisher’s exact test was performed in cases where the expected value count was <5. The differences in means, medians, and frequency distributions were statistically significant if the *p*-value was <0.05. The results are reported using mean values with a ±SD (standard deviation), median values with a ±IQR (interquartile range), and frequency tables. The variables influencing treatment failure risk in both the surgery group and the MMA embolization group were calculated using binary logistic regression with treatment failure as a dependent variable.

## 3. Results

### 3.1. Baseline Characteristics of the Surgical Treatment Group and MMA Embolization Group; Clinical and Radiological Findings

One hundred forty-two cases of unilateral or bilateral chronic subdural hematoma (CSDH) were included in the study. Seventy-eight cases (54.9%) were treated surgically and MMA embolization with PVA particles was performed in 64 cases (45.1%). We included 58 (74.4%) primary hematoma cases in the surgical group and 54 (84.4%) primary hematoma cases in the MMA group. Four cases (5.1%) of recurrent hematoma treated primarily with surgery were included in the surgery group, and nine (14.1%) cases where recurrences were treated primarily with surgery were placed into the MMA group. Fourteen cases (14.9%) of recurrent hematomas treated primarily with MMA embolization were included in the surgery group, and none were included in the MMA group. We observed significant differences in the frequency distribution due to the distribution of recurrences in cases primarily treated with MMA embolization between both groups (*p* = 0.002).

According to patient symptoms at admission, we observed a significant difference in frequency distribution between the surgery and MMA group (*p* = 0.001). There was no statistical difference in frequency distribution between the groups in relation to anticoagulation and antiplatelet medication in the patient’s history (*p* = 0.439). 

The mean hematoma width was 21 mm (±5) in the surgical group and 18 mm (±6) in the MMA embolization group (*p* = 0.001). The mean midline shift was 8 mm (±5) in the surgical group and 5 mm (±4) in the MMA embolization group (*p* = 0.001). All baseline characteristics are summarized in Table 1. 

### 3.2. Treatment Results—Surgical Group and MMA Embolization Group; Clinical and Radiological Findings

Treatment failure was observed in 18 cases (23.1%) in the surgery group and 14 cases (21.9%) in the MMA embolization group (*p* = 0.865). In the surgery group, revision surgery due to primary treatment failure was performed in nine cases (11.5%); in the MMA embolization group, revision surgery was necessary in 14 cases (21.9%). After primary treatment failure, we performed the second treatment using MMA embolization in 9 cases (11.5%) in the surgery group and 0 (0.0%) cases in the MMA embolization group (*p* = 0.006).

The mean follow-up duration was 44 days (±55) in the surgery group and 72 days (±76) in the MMA embolization group (*p* = 0.015). The treatment results are summarized in Table 2.

### 3.3. Comparison of Treatment Complications—Surgical Treatment Group and MMA Embolization Group

Recurrence of the subdural hematoma was observed in 12 cases (15.4%) in the surgery group and 4 cases (6.3%) in the MMA embolization group (*p* = 0.087). Wound infection occurred in 2 cases (2.6%) in the surgery group and 0 cases (0.0%) in the MMA embolization group (*p* = 0.197). Periprocedural complications in relation to MMA embolization were amaurosis in one case (1.6%) (*p* = 0.268) and facial nerve palsy in two cases (3.1%) (*p* = 0.116%). 

Neurological deterioration with a need for a second treatment was observed in 11 cases (17.2%) in the MMA embolization group and no cases in the surgery group (*p* = 0.001).

In each group, we documented non-procedural-related complications. One case of a stroke (1.3% in surgery, 1.6% MMA embolization group, *p* = 0.888) and one death (1.3% in surgery, 1.6% MMA embolization group, *p* = 0.888) were recorded. Treatment-related complications are summarized in Table 3.

### 3.4. Variables Predicting Treatment Failure—MMA Embolization Group

The binary logistic regression model in the MMA embolization group with treatment failure as the dependent variable was significant (χ^2^ = 20.391, *p* = 0.005, *n* = 48). However, 16 cases were excluded due to bilateral hematoma because the variable midline shift of the bilateral hematoma was not taken into account. Higher midline shift was identified as a factor predicting treatment failure (OR 1.331, *p* = 0.025). An even stronger predictive value was found for the presence of acute (hyperdense) hematoma on the admission CT scan (OR 10.960, *p* = 0.034). Patient age, severity of symptoms, anticoagulation or antiplatelet medication in patient history, hematoma width, and membranous segmentation of the hematoma had no importance in the prediction of treatment failure. Nagelkerke R square was 0.513, which corresponds to a strong effect according to Cohen’s effect size (Table 4).

### 3.5. Variables Predicting Treatment Failure—Surgical Group

In the surgical group, the binary logistic regression model was significant (χ^2^ = 17.906, *p* = 0.012, *n* = 60) as well. Eighteen cases were excluded due to bilateral hematoma, where the variable midline shift was not taken into account. Also in the surgical group, the midline shift predicted treatment failure (OR 1.319, *p* = 0.047). A significantly stronger predictive value in relation to treatment failure was found for a membranous segmentation of the hematoma on admission CT scan (OR 12.072, *p* = 0.042). On the other hand, the hyperdense parts of the hematoma, hematoma width, anticoagulation or antiplatelet medication, severity of symptoms, and age of the patient had no importance for the prediction of treatment failure. Nagelkerke R square was 0.397, which corresponds to a strong effect according to Cohen’s effect size (Table 5).

### 3.6. Comparison of Treatment Results in the Surgery Alone versus Concomitant MMA Embolization and Surgery Subgroups

The surgery group data were divided into a subgroup of 58 cases (80%) treated primarily with surgery and 14 cases (20%) treated by MMA embolization with concomitant surgery due to secondary neurological deterioration. In the primary surgical subgroup, we reported 13 treatment failure cases (22%); in the concomitant MMA embolization and surgery group, we observed two treatment failure cases (14%). However, this difference in treatment failure rate was not statistically significant (Fisher’s exact test, *p* = 0.719). 

## 4. Discussion

We found differences in the baseline characteristics between the surgery and MMA embolization groups. As expected, we observed higher rates of major symptoms and higher NIHSS scores in the surgery group. The mean midline shift and the mean hematoma width were larger as well. Due to more severe symptoms and a slightly wider hematoma, rapid evacuation of the hematoma was necessary. However, our criteria for surgical treatment correlated with the criteria mentioned in other trials, particularly hematoma width > 10 mm, midline shift > 5–7 mm, and evidence of neurological deficits [1,5,9,17]. In cases of secondary surgical treatment of hematoma recurrences, we observed significant differences in the frequency distribution between surgery and MMA embolization groups. This was attributed to neurological deterioration in 11 patients a few days after the MMA embolization. In these cases, concomitant surgery and hematoma evacuation due to the hematoma mass effect were necessary. It is assumed that this combined treatment could reduce the risk of recurrent hematoma compared to surgery alone [4,14,15]. Our results mirrored these findings, but with 14% of cases showing treatment failure in combined endovascular and surgical treatment versus 22% of cases with surgical treatment alone. The difference was not statistically significant.

The primary outcome in the surgery and MMA embolization groups was statistically comparable, with 23.1% treatment failure cases in surgery and 21.9% in the MMA group. At first glance, these results do not correspond with the findings in the literature, where the endovascular treatment recurrence rate ranges from 1.4% to 8.9% [4,18,19,20]. However, an exact comparison here is not possible because of the differences in primary outcome definitions between the trials. In this study, we defined the primary outcome—treatment failure—as a need for secondary treatment of the hematoma, not just due to recurrence, but also in cases of postoperative bleeding complications or early neurological deterioration. Recurrent hematoma alone was observed in 6.3% of cases in the MMA embolization group and 15.4% of cases in the surgery group, which corresponds to the studies mentioned above, as well as the recurrence rates of surgical treatment mentioned in other trials [12,13]. 

In the MMA embolization group, we observed a significantly higher rate of neurological deterioration with a need for subsequent surgery. In all 11 cases, neurological worsening occurred within 72 h. We assumed these complications were caused by an incorrect indication for MMA embolization rather than by a failure of the MMA embolization procedure. The analysis of these MMA treatment failure cases showed a higher mean midline shift of 8 mm (±4) and hematoma width of 21 mm (±4), matching the mean values of the surgery group. Within the context of mean maximal hematoma width and mean midline shift in the MMA embolization group, these findings could suggest a hematoma width of less than 18 mm and a midline shift of less than 5 mm are safe criteria for MMA embolization. However, in cases of asymptomatic patients with a midline shift of less than 5 mm, conservative therapy could be taken into account as well [8]. 

Concerning treatment complications, we found procedure-specific complications in both groups. In the MMA embolization group, we had a case of unilateral amaurosis probably caused by iatrogenic PVA particle backflush and embolization of the central retinal artery anastomosed to the MMA branch via the lacrimal artery to the ophthalmic artery [21,22,23]. Therefore, during the procedure, it is necessary to actively look for these anastomoses and perform the particle embolization slowly under controlled hypertension to reach a flow reversal in case of the presence of anastomosis or choose another MMA embolization technique either with liquid embolic agents or with coils. We also reported two cases of transient unilateral peripheral facial nerve palsy, probably as a result of iatrogenic embolization of the stylomastoid artery via anastomosis with MMA [24]. Further, in the surgery group, we observed two cases (2.6%) of surgical site infection, which corresponds with the data in the literature with the infection rate ranging from 1.9% to 15.3% [25,26,27]. In both groups, there were statistically comparable periprocedural bleeding complications, and in each group, there was one case of stroke and one case of death not related to the procedure.

Looking for variables predicting treatment failure, we found that in both the surgery and MMA embolization groups, a higher midline shift was a weak risk factor for treatment failure. Kim et al. (2017) presented similar conclusions and suggested anticoagulation therapy as a risk factor [3]. In our study, anticoagulation or antiplatelet treatment in the patient’s medical history did not have any predictive value. The presence of membranous segmentation of a CSDH on an admission CT scan increased the odds 12 times for treatment failure in the surgery group. On the other hand, the presence of acute and subacute (hyperdense) parts in the CSDH increased the odds almost 11 times for treatment failure in the MMA embolization group and was a strong predictor as well.

This clinical study had several limitations. The first is the retrospective nature of the research with the corresponding biases that can be associated with this study design. We also acknowledge some statistical variances in the baseline characteristics of both groups. Another limitation is the monocentric characteristics of the study and the relatively small cohort of patients. Therefore, our results should be verified by future studies. 

## 5. Conclusions

MMA embolization and CSDH evacuation should not be seen as competing, but as complementary treatment modalities. Our results could help create correct indication criteria for hematoma treatment, especially for patients with no or mild neurological symptoms. Those with a maximal hematoma width < 18 mm and midline shift < 5 mm measured on admission CT scan in the frontal plane without an acute or subacute (hyperdense) hematoma could be candidates for endovascular treatment with MMA embolization. Those with a maximal hematoma width > 18 mm and midline shift > 5 mm measured on admission CT scan in the frontal plane without membranous hematoma segmentation could benefit from surgical hematoma evacuation and a closed drainage system.

## Figures and Tables

**Table 1:** Baseline characteristics of the surgical treatment group and MMA embolization group; Clinical and radiological findings.

		Surgery	MMA Embolization	*p*-Value
		*n* = 78 (54.9%)	*n* = 64 (45.1%)	
mean age in years (SD)		78 (10)	75 (12)	0.065 *
female sex (%)		18 (23.1%)	16 (25%)	0.845
median NIHSS score on admission (IQR)		2 (3)	1 (2)	0.001 **
Symptoms (%)	none	1 (1.3%)	5 (7.8%)	0.001
	minor	21 (26.9%)	32 (50%)	
	major	56 (71.8%)	27 (42.2%)	
anticoagulation/antiplatelet therapy (%)	none	37 (47.4%)	28 (43.8%)	0.439
	anticoagulation	21 (26.9%)	19 (29.7%)	
	antiplatelet monotherapy	20 (25.6%)	15 (23.4%)	
	dual antiplatelet therapy	0 (0.0%)	2 (3.1%)	
hyperdense (acute) parts of hematoma on CT scan (%)		30 (38.5%)	19 (29.7%)	0.274
segmentation of hematoma, membranes on CT scan (%)		43 (55.1%)	39 (60.9%)	0.486
mean hematoma width in mm (SD)		21 (5)	18 (6)	0.001 *
mean midline shift in mm (SD)		8 (5)	5 (4)	0.001 *
secondary treatment of hematoma recurrence (%)	none	58 (74.4%)	54 (84.4%)	0.002
	surgery	4 (5.1%)	9 (14.1%)	
	embolization	14 (17.9%)	0 (0.0%)	
	both	2 (2.6%)	1 (1.6%)	
subdural hematoma site (%)	left	30 (38.5%)	28 (43.8%)	0.549
	right	30 (38.5%)	19 (29.7%)	
	both	18 (23.1%)	17 (26.6%)	

For the marked values (*), the independent sample T-test and Mann–Whitney U test (**) were used. For other cases, χ^2^-test and Fisher’s exact test were used; 2-sided *p*-value is presented. MMA = middle meningeal artery; IQR = interquartile range; NIHSS = National Institutes of Health Stroke Scale; SD = standard deviation; minor symptoms = headache, vertigo, dizziness; major symptoms = hemiparesis, dysphasia, decreased consciousness.

**Table 2:** Comparison of the treatment results—surgical treatment group and MMA embolization group.

		Surgery	MMA Embolization	*p*-Value
		*n* = 78 (54.9%)	*n* = 64 (45.1%)	
treatment failure (%)		18 (23.1%)	14 (21.9%)	0.865
secondary treatment of the treatment failure cases (%)	no	46 (59.0%)	31 (48.4%)	0.006
	surgery	9 (11.5%)	14 (21.9%)	
	AMM embolization	9 (11.5%)	0 (0.0%)	
	follow-up loss	14 (17.9%)	19 (29.7%)	
median number of CT scans during follow-up (IQR)		2 (1)	2 (1)	0.670 **
mean follow-up in days (SD)		44 (55)	72 (76)	0.015 *

For the marked values (*), the independent sample T-test and Mann–Whitney U test (**) were used. For other cases, χ^2^-test and Fisher’s exact test were used; 2-sided *p*-value is presented. MMA = middle meningeal artery; IQR = interquartile range; SD = standard deviation.

**Table 3:** Comparison of the treatment-related complications—surgical treatment group and MMA embolization group.

	Surgery	MMA Embolization	*p*-Value
	*n* = 78 (54.9%)	*n* = 64 (45.1%)	
recurrent hematoma (%)	12 (15.4%)	4 (6.3%)	0.087
acute hematoma bleeding (%)	6 (7.7%)	4 (6.3%)	0.738
wound infection (%)	2 (2.6%)	0 (0%)	0.197
amaurosis (%)	0 (0%)	1 (1.6%)	0.268
neurological deterioration (%)	0 (0%)	11 (17.2%)	0.001
stroke (%)	1 (1.3%)	1 (1.6%)	0.888
death (%)	1 (1.3%)	1 (1.6%)	0.888
facial nerve paresis (%)	0 (0%)	2 (3.1%)	0.116

χ^2^-test and Fisher’s exact test were used; 2-sided *p*-value is presented. MMA = middle meningeal artery.

**Table 4:** Variables predicting treatment failure—MMA embolization group.

Variables	OR (95% CI for OR)	*p*-Value
Age	1.047 (0.960–1.143)	0.300
Severity of symptoms	2.698 (0.408–17.858)	0.303
Anticoagulation or antiplatelet therapy	1.606 (0.241–10.712)	0.625
Hematoma width	1.238 (0.960–1.597)	0.101
**Midline shift**	**1.331** (**1.037–1.707**)	**0.025**
Segmentation of hematoma with membranes	0.249 (0.030–2.092)	0.200
**Hyperdense (acute) parts of hematoma**	**10.960** (**1.200–100.129**)	**0.034**

Dependent variable treatment failure entering the binary logistic regression model. Values marked in bold are significantly different at *p* < 0.05. CI = confidence interval; OR = odds ratio.

**Table 5:** Variables predicting the treatment failure—surgery group.

Variables	OR (95% CI for OR)	*p*-Value
Age	1.042 (0.958–1.132)	0.335
Severity of symptoms	7.506 (0.575–97.972)	0.124
Anticoagulation or antiplatelet therapy	2.189 (0.420–11.398)	0.352
Hematoma width	0.887 (0.737–1.067)	0.204
**Midline shift**	**1.319 (1.004–1.732)**	**0.047**
**Segmentation of hematoma with membranes**	**12.072 (1.091–133.570)**	**0.042**
Hyperdense (acute) parts of hematoma	0.438 (0.077–2.498)	0.352

Dependent variable treatment failure entering the binary logistic regression model. Values marked in bold are significantly different at *p* < 0.05. CI = confidence interval; OR = odds ratio.

## Data Availability

The data presented in this study are available on request from the corresponding author.
